# Silhouette Lymphangioma: An Unknown Macular Form of Cutaneous Lymphangioma

**DOI:** 10.34172/aim.2022.75

**Published:** 2022-07-01

**Authors:** Betul Tas, Seyda Andac, Aysel Caglar

**Affiliations:** ^1^Department of Dermatology and Venereology, Bagcilar Research and Training Hospital, University of Health Sciences, Istanbul, Turkey; ^2^Department of Radiodiagnostics, Bagcilar Research and Training Hospital, University of Health Sciences, Istanbul, Turkey; ^3^Department of Dermatopathology, Bagcilar Research and Training Hospital, University of Health Sciences, Istanbul, Turkey

**Keywords:** Lymphangioma, Lymphatic vessel tumors, Skin abnormalities

## Abstract

Unusual angiomatous or lymphangiomatous vascular malformations are rarely seen. One of them is lymphangioma (LA), which is a rare benign lymphovascular abnormality. LA is usually seen in the types of circumscriptum (or capillary), cavernous and cystic. Here, we report a unique case of LA with a patchy appearance. The patient presented due to unusual symptoms and eccentric clinical manifestation of the lesion. Here, we present a new lymphatic entity which was diagnosed as LA with its clinical, radiological and pathological findings. Written informed consent of the patient was obtained for this report. To the best of our knowledge this macular form of cutaneous LA has not been previously reported in literature. Macular LA should be kept in mind when faced with a colored long-term macular lesion on the skin.

## Introduction

 Lymphangioma (LA) is a rare and benign hamartoma of the lymphovascular system which involves the skin and subcutaneous tissues. It occurs in 4% of all vascular tumors and approximately ¼ of benign vascular malformations in the children.^1^ LAs are mostly seen at birth and in nevoid, superficial or deeper patterns.^2^ They are usually classified in two major groups based on the depth and size of the lymphatics. Superficial vesicular lesions are called lymphangioma circumscriptum (LC), whereas the deeper group includes cavernous LA and cystic hygroma.^3,4^ Vascular hamartomas may appear in unusual and overlapping forms such as hemato-lymphangiomas.^5^ hyperkeratotic angiomas (angiokeratoma)^6^ or hyperkeratotic lymphangiomas (lymph angiokeratoma).^7^ Here, we report a unique and unknown form of LA with an unusual patchy appearance.

## Case Report

**Figure 1:**
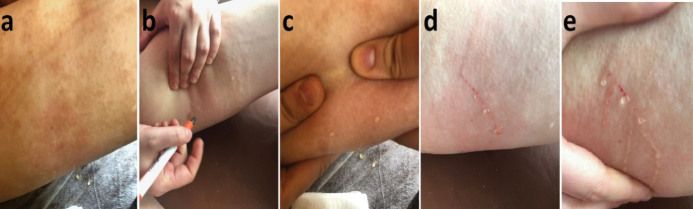
Appearance of multiple clear droplets on the surface of the brownish patch due to scratching the lesion. a) Macular appearance of the lesion. b-e) Transparent droplets oozing from scratching the lesion

**Figure 2:**
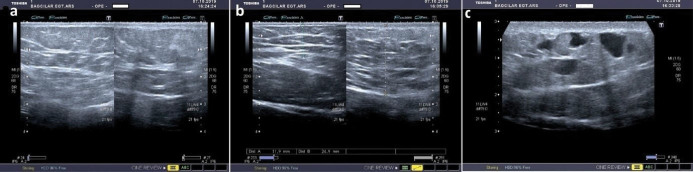
Axial Ultrasound Images Showing the Thickness of the Epidermis. (a) On the right, the epidermis on lesion site is thicker than the normal epidermis on the left side. (b) On axial ultrasound images, it is seen that the entire thickness of the skin, from the epidermis to the muscle, is increased at the lesion’s location compared to the normal area. (c) In the ultrasound image, the lesion is ill-defined, and thin-walled, dilated and tortuous vascular components are seen in the estimated lesion area

**Figure 3:**
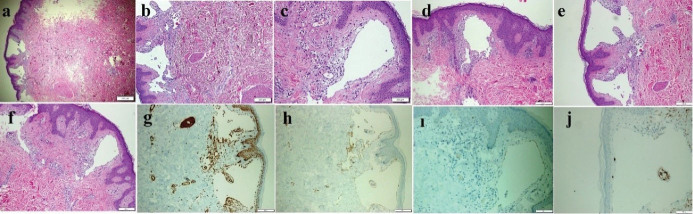
Thin-Walled Dilated Vascular Proliferations Coated with a Single-Row Endothelium and Scattered Lymphocytes in the Stroma Mainly, in the Papillary Dermis and Also Focal Areas in the Reticular Dermis. (a-f) Some of the endothelial cells have hobnail features. Positive immunohistochemical staining of vascular endothelial cells for SMA (g), CD31(h), and D2-40 (i), but not for CD34 (j) (a: HE & X40, b-f: HE& X100, g-i: X100 and j: X200)

 An 18-year-old girl was admitted to our institution seeking a cosmetic remedy for her bizarre leg lesion, which had been noticed since she was ten years old. She stated that it was initially a light-brown and asymptomatic macula, but then darkened after the age of 13. The patient complained of both its ugly color and a leak wetting her clothes which occurs when the lesion rubs anywhere or any pressure was applied on it. The personal and family history of the patient was unremarkable. On dermatological examination, a brownish, smooth-surfaced, patchy irregular-bordered, macular plaque was seen on the inner surface of the left leg approximately 23 × 18 cm in diameter. The surface of the lesion was dry unless pressure was applied, and it did not blanch significantly on slight pressure. When the lesion was strongly scratched with a blunt-tip pen, multiple, small, clear droplets appeared on the surface of the brownish patch (Figure 1a-e). When more pressure was applied to the lesion, abundant liquid sprayed gushing from many foci resembling a fountain. The rest of the dermatological and physical examinations, and routine laboratory investigations did not show any additional pathology. Fifteen mL of the flowing liquid was taken in a sterile tube within approximately 15 minutes. Microbiological analysis of the liquid was sterile. Biochemical analysis showed; sugar, 100.8 mg/dL; urea, 22.2 mg/dL; nitrogen, 32.5 mg/dL; creatinine, 1.5 mg/dL; chloride 110.7 mmol/L; sodium 141.6 mmol/L; phosphorus, 116 mg/dL; inorganic phosphorus, 5.5 mmol/L; calcium 9.7 mg/dL; potassium 5.03 mmol/L; total protein 2.91 mg/dL; triglyceride, 160 mg/dL; and LDH 192U/L. The liquid contained abundant lymphocytes. Sonographic examination demonstrated a heterogeneous mainly hyper-isoechoic, ill-defined intradermal lesion. Compared with the same side of the opposite extremity, the epidermis was thickened at the site of the lesion, and was wider then the surrounding normal tissue (Figure 2a). Additionally, the thickness the entire skin was increased at the lesion site compared to normal areas (Figure 2b). Thin-walled dilated and tortuous vascular components were seen in the lesion (Figure 2c). However, the vasculature showed no color codes on color Doppler ultrasonography examination, probably because of the low velocity of the lymphatic vessels’ flow, which was below the resolution of the imaging system. Whole abdominal ultrasonography was normal. Magnetic resonance imaging of the left leg and brain did not show any deep or inner component. An excisional biopsy was taken from the middle of the lesion. Histopathological examination showed thin-walled dilated vascular proliferations which were coated with a single row of endothelium and scattered lymphocytes in the stroma, mainly in the papillary dermis and also focal areas in the reticular dermis. Some of the endothelial cells had hobnail features (Figure 3a-f). Immunohistochemically, the vascular endothelial cells stained positive for SMA (smooth muscle actin), CD3, and D2-40, but not for CD34 (Figures 3g, 3h, 3i, and 3j, respectively). With these findings, the lesion was diagnosed as LA. Because of its unusual macular appearance, we preferred to describe it as “silhouette LA”. Er:YAG laser ablation was recommended for treatment of the lesion, however, the patient did not accept the therapy because of its possible scarring effects. Thus, we started thrice weekly topical imiquimod 5% therapy on the lesions. The patient is in the 16^th^ week of the therapy, and still being followed. However, no symptomatic or cosmetic improvement has been observed yet.

## Discussion

 Classical types of LA include the capillary, cavernous and cystic forms. However, it can be difficult to make a diagnosis based on clinical appearance alone, when faced with an unusual form of the lesions. This difficulty may originate from the lesions either not being known, or, having unusual appearances, location or histopathologic features. LC is the most common form of cutaneous LAs. Clinically, itis seen as clustered (localized) or diffuse (classical) translucent vesicles approximately 1-5 mm in diameter, filled with clear fluid (lymph). Congenital forms mostly appear early in life usually at birth. LAs arise as a result of a secondary dilatation of superficial lymphatics, due to the pressure caused by the malformations of deep dermal and subcutaneous lymphatics.^8^ Localized LAs are seen more rarely, which appear as small, clear lesions. Although they can occur at any age or any site of the body, the neck, axillary folds, shoulders, proximal parts of the extremities, and tongue are the most frequently affected areas.^2,3^ Cystic LAs are composed of subcutaneous nodules which are usually painless. Cystic hygroma is a prototype of subcutaneous LAs, which manifests as a large cystic mass. It is seen at birth or in early infancy with a predilection for the neck, axillary and inguinal regions.^2^ The main histopathological findings of LC are enlarged lymphatic vessels in the papillary dermis, which are usually situated just beneath the epidermis, but they can also extend to the reticular dermis. They are coated by flat, discontinuous endothelial cells.^2,8^ In the stroma, scattered lymphocytes may be seen.^8^ In the cystic forms, ectatic, irregular, and interconnected lymphatics are seen in the subcutaneous fat, some of which may contain smooth muscle bundles.^2^ The lymphatic endothelial cells of LAs are positive for CD31 and D2-40.^8^ CD31 is a common marker for vessel endothelium. Its positivity is independent of lymphatic or angiomatous origin of a vessel, whereas D2-40 is a sensitive and relatively specific marker for lymphatic endothelium, but is not expressed in the blood vessels. On the other hand, lymphatics are usually negative for CD34, even though the staining can be irregularly obtained positive for some of them. However, its intensity is very weak in the lymphatics when compared to D2-40.^9^ The lymphatic lesion of our patient was absent at birth, and was noticed when she was 10 years old. Histopathologically, it was localized mainly in the papillary dermis and there were focal and smaller ectatic lymphatics in the reticular areas. They stained strongly positive for CD31 and D2-40, but not for CD34. So, the lesion was judged to be a lymphatic malformation. Although histologically, it was a lymphangioma, it contained neither LC-like protruding vesicular components on the skin, nor a cavernous lymphangioma-like subcutaneous component. In the therapy of a localized LA, different treatment options have been suggested such as cryosurgery, electrocautery, laser ablation, sclerotherapy, superficial radiotherapy, and surgical excision.^3,8,10^ However, superficial ablative methods may cause recurrence of the lesion, when a possible deep component is not taken into account. Conversely, unnecessarily deep interventions may lead to unwanted scars.^11,12^ Recently, topical imiquimod application has been recommended in the treatment of LCs.^13,14^ Because of our patient’s refusal of an invasive therapy, we began to treat the lesion with topical imiquimod. Although the present case was histopathologically a superficial cutaneous lymphatic malformation, it was not similar to any usual clinical variants of LA. In the pathogenesis of LC development, Whimster et alhypothesized that during embryogenesis, abnormal lymphatic cisterns grow independently from normal lymphatics in the deep subcutaneous tissue. With the contraction of smooth muscle cells which line these cisterns, the lymphatics enlarge and then protrude through the skin to form clear vesicles on the skin surface.^15,16^ Martinez-Menchon et alsupported this hypothesis by their imaging studies in 2004.They showed that multi-lobular big cisterns of LC were located in the deep dermis, and were not related with adjacent normal lymphatics.^17^ Additionally, some external or internal factors such as trauma, friction, or hormones have been blamed as possible triggers for the acquired development of a LC, by causing transformation, proliferation, and migration towards upper layers of the pluripotent rudimentary endothelial cells.^15-17^ We believe that the present lesion may have been caused by inadequate or slow effect of these acquired triggers, under the influence of individual biogenetic mechanisms. It is also thought that the lesion might be a late-onset lymphangiomatous equivalent of a nevus flammeus, because of their similar clinical and histologic properties. Nevertheless, the development of late vesicles at the surface of the lesion can be expected in advanced age. In differential diagnosis, discolored skin patches such as pigmented nevi, café-au-lait spots, Mongolian spots, post-inflammatory hyperpigmentation, fix drug eruption, and segmental pigmentation disorders should be considered.

 In conclusion, silhouette LA is an unknown and new entity in the category of superficial lymphangioma, which has not been included in the available textbooks until now. It should be kept in mind when faced with a dark-colored long-term macular lesion on the skin, so that patients are not exposed to unnecessary treatments.
